# Frailty and transplant-free survival of patients with liver cirrhosis: A meta-analysis

**DOI:** 10.1371/journal.pone.0302836

**Published:** 2024-05-09

**Authors:** Chunhui Yuan, Weihua Li, Jie Liu, Jianguo Li

**Affiliations:** Department of Gastroenterology, Changsha Fourth Hospital, Changsha, Hunan Province, China; Institute for Clinical and Experimental Medicine, CZECH REPUBLIC

## Abstract

**Background:**

Frailty is a common condition among patients with liver cirrhosis. Nonetheless, its role in predicting liver transplant-free survival (TFS) remains unclear.

**Aim:**

This systematic review and meta-analysis were conducted to elucidate the relationship between frailty and TFS in patients with cirrhosis.

**Methods:**

Cohort studies addressing the objective of this meta-analysis were extracted from PubMed, Embase, and Web of Science databases. Between-study heterogeneity was assessed with the Cochrane Q test, and the I^2 statistic was estimated. Random-effect models, considering potential heterogeneity, were employed to combine the results.

**Results:**

The meta-analysis encompassed 17 cohort studies involving 6273 patients with cirrhosis, of whom 1983 (31.6%) were classified as frail at baseline. The follow-up periods in the included studies ranged from 3 to 29 months, with an average duration of 11.5 months. The analysis revealed that frailty was significantly associated with a poor TFS (risk ratio [RR]: 2.07, 95% confidence interval: 1.72 to 2.50, *p*<0.001; *I*^*2*^ = 51%). Sensitivity analyses that sequentially omitted one dataset consistently supported these findings (RR: 1.95 to 2.17, *p*<0.05 in all cases). Subgroup analyses based on variables such as study design, mean age of patients, baseline Model for End-Stage Liver Disease score, tool used for frailty evaluation, follow-up duration, and study quality score also yielded congruent results.

**Conclusions:**

The evidence suggests that frailty may be an independent risk factor for poor TFS in patients with liver cirrhosis, thus emphasizing the importance of early identification and management of frailty in this population.

## Introduction

Liver cirrhosis is a prevalent manifestation of advanced liver diseases, encompassing chronic viral hepatitis, alcoholic liver disease, and non-alcoholic fatty liver disease [1,2]. This condition detrimentally affects various aspects of health-related quality of life, including mental well-being and physical factors, while also diminishing the functional capacity of affected individuals [3,4]. On a global scale, cirrhosis is responsible for more than 2 million fatalities annually [5,6]. Currently, no evidence-based pharmacological interventions are capable of reversing the fibrosis that leads to cirrhosis [7]. Consequently, liver transplantation remains the sole curative alternative for individuals in the end-stage of cirrhosis [8]. Given the multifactorial and intricate pathogenesis of liver cirrhosis, it is reasonable to explore innovative approaches for risk stratification in cirrhosis patients. In the clinical realm, frailty has emerged as a potential factor within the framework of comprehensive geriatric assessment, denoting a state of cumulative deterioration across various physiological systems and heightened susceptibility to unfavorable outcomes [9,10]. Notably, frailty has been demonstrated as a substantial prognosticator of overall mortality in elderly individuals [11]. Subsequent analysis indicates that in addition to the older community population, frailty may also serve as a risk factor for poor prognosis in patients with diverse chronic diseases, including cardiovascular diseases [12] and cancer [13]. Earlier studies have demonstrated the prevalence of frailty in patients with liver cirrhosis, ranging from 17% to 43% [14]. However, it remains uncertain whether frailty can accurately predict the clinical outcome of patients with cirrhosis [15]. Consequently, we conducted a systematic review and meta-analysis to examine the potential correlation between frailty and liver transplant-free survival (TFS) in patients with cirrhosis.

## Materials and methods

Throughout the process of planning, conducting, and reporting the study, the Preferred Reporting Items for Systematic Reviews and Meta-Analyses statement [16] and Cochrane Handbook [17] were followed.

### Search of databases

We searched electronic databases, including PubMed, Embase, and Web of Science, starting inception and ending July 22, 2023, for studies published by that date. The search was performed with the terms including (1) "frailty" OR "frail"; and (2) "cirrhosis" OR "cirrhotic" OR "liver fibrosis" OR "hepatic fibrosis." Only human studies published in English as full-length articles in peer-reviewed journals were considered. As part of our manual screening process, references from relevant original and review articles were screened for possible relevant studies.

### Inclusion and exclusion criteria of studies

Inclusion criteria were developed per the PICOS recommendations and according to the aim of the meta-analysis.

P (patients): Adult patients (18 years or older) with a confirmed diagnosis of liver cirrhosis.

I (exposure): Patients with frailty at baseline. Methods for evaluating frailty were consistent with those of the original studies.

C (control): Patients without frailty at baseline.

O (outcomes): The incidence of liver transplant-free survival (TFS) compared between cirrhotic patients with and without frailty at baseline, with a follow-up duration of at least three months. The TFS was defined as being alive without having undergone a liver transplant, and it was selected as the outcome of the meta-analysis because this is an essential hard clinical outcome for patients with liver cirrhosis, which have been frequently used in previous high-quality clinical trials of liver cirrhosis [18–20].

S (study design): Cohort studies, which included prospective and retrospective cohort studies.

Reviews, editorials, studies that included other liver diseases rather than cirrhosis, and studies that did not evaluate frailty or did not report the outcome of interest were excluded. Studies with a follow-up duration of <3 months were also excluded because we did not want to investigate the acute influence of frailty on the clinical outcome of patients with cirrhosis. In cases of overlap in patient populations, the study with the largest sample size was included in the meta-analysis.

### Data extraction and quality evaluation

The two authors carried out literature searches, data collection, and study quality assessments independently. In case of discrepancies, a third author was contacted for a discussion to reach a consensus. Among the studies included in the analysis, we collected information regarding study information, design characteristics, diagnosis of the patients, demographic factors, severity of the disease (model for end-stage liver disease [MELD] score), evaluating scale for frailty, number of patients with frailty in each study, follow-up durations, and variables adjusted when the association between frailty and TFS of patients with cirrhosis were evaluated. In terms of quality, the study was scored using the Newcastle–Ottawa Scale [21] based on the criteria for participant selection, the comparability of the groups, and the validity of the outcomes. Nine stars were on the scale, with a larger number representing a better study.

### Statistics

Risk ratios (RRs) corresponding to a 95% confidence interval (CI) were used as the variables to indicate the association between frailty and liver transplant-free mortality of patients with cirrhosis. An RR>1 indicates a worse TFS in patients with frailty at baseline. A logarithmical transformation was performed on the RR and its corresponding stand error (SE) from each study to stabilize and normalize its variance [22]. In order to estimate between-study heterogeneity, the Cochrane Q test and the I^2^ statistic [23] were used. An *I*^*2*^>50% indicates that there is significant heterogeneity between studies. A random-effects model was applied to pool the results because this model has been considered to incorporate the influence of potential heterogeneity [17]. Sensitivity analyses excluded one dataset at a time to evaluate how individual studies affected meta-analysis results [24]. In order to determine the influence of study characteristics on the outcome, subgroup analyses were performed according to the study design, the mean age of the patients, average MELD score at baseline, evaluating scale for frailty, follow-up duration, and study quality scores. Subgroups were defined based on the medians of continuous variables. A funnel plot is used to estimate publication bias based on visual symmetry judgments, along with Egger’s regression asymmetry test [25]. The statistical analyses were carried out with RevMan (Version 5.1; Cochrane Collaboration, Oxford, UK) and Stata software (version 12.0; Stata Corporation, College Station, TX).

## Results

### Database search and study retrieval

**Fig 1:**
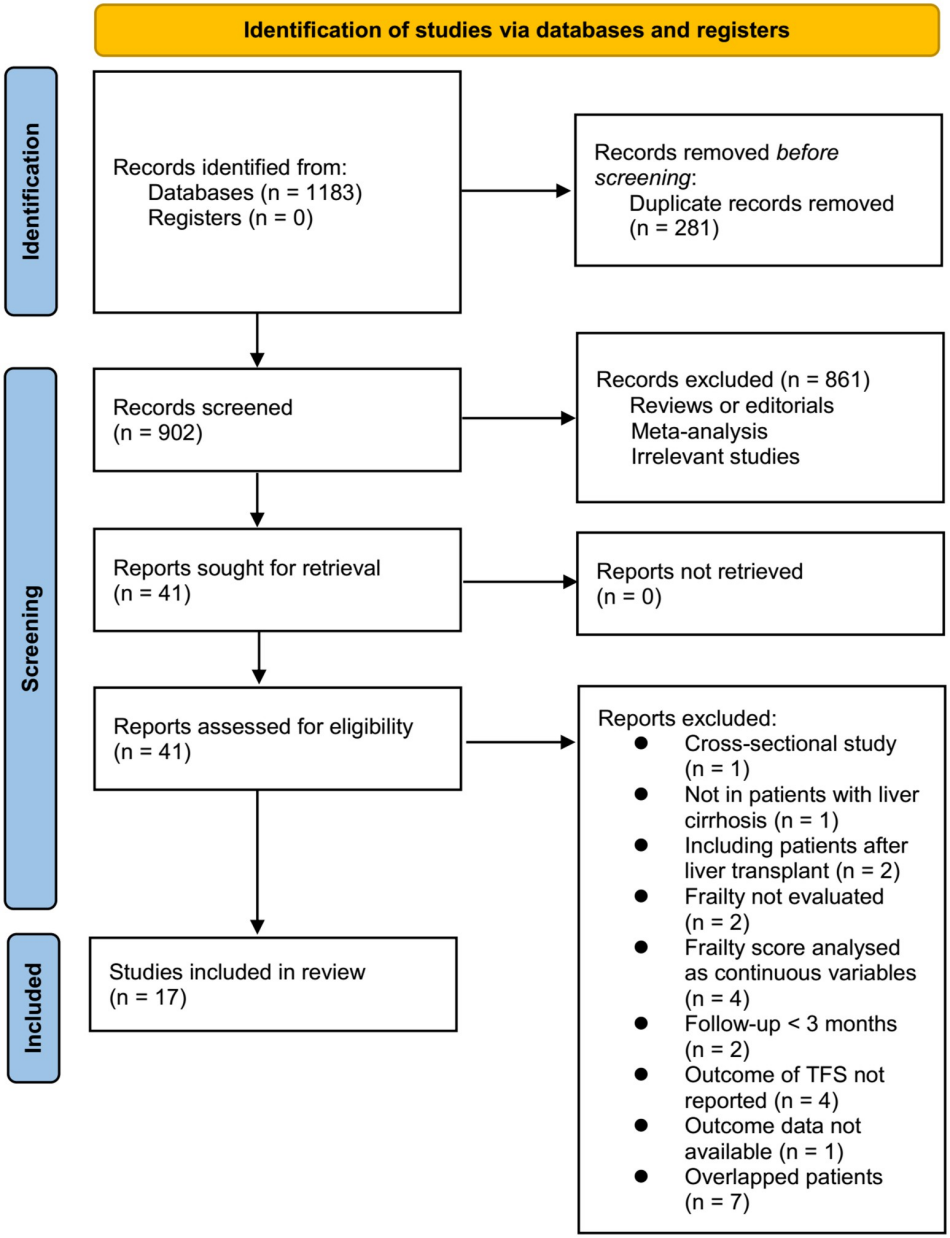
Flowchart of database search and study inclusion.

Fig 1 illustrates the methodical process of literature search and study selection. Initially, 1183 records were retrieved from the database search, and 281 duplicates were subsequently eliminated. Following the title and abstract screening, 861 studies were excluded as they did not align with the objectives of the meta-analysis. A comprehensive full-text review of 41 studies was then conducted, excluding 24 studies for the reasons specified in Fig 1. Consequently, 17 studies met the criteria and were included in the subsequent meta-analysis [26–42].

### Study characteristics

The characteristics of the included studies are summarized in Table 1. These studies were published between 2015 and 2023 and performed in the United States, China, Germany, Chile, Slovakia, Spain, Thailand, Canada, and India. As for the study design, were prospective cohort studies [27–30,32–36,39–42], and four were retrospective cohort studies [26,31,37,38]. A total of 6273 patients with cirrhosis were included in the meta-analysis. The sample size of the included studies varied from 116 to 1044. The mean ages of the included patients were 50.2 to 64.0 years, with the proportions of men varying from 46.2% to 96.6%. The average MELD score of the included patients was 9 to 21 at baseline. Regarding the evaluating tool for frailty, the Liver Frailty Index (LFI) was used in nine studies [27,30,33–35,37,39–41], whereas for the other studies, the Activities of Daily Living scale [26], the Carolina Frailty Index [28,38], the Clinical Frailty Scale [29], the Fried Frailty Index [32,36,42], and the Hospital Frailty Risk Score [31] were used respectively. Accordingly, 1983 (31.6%) of the included patients had frailty at baseline. The follow-up durations of the included studies were 3 to 29 months (mean: 11.5 months). Multivariate regression analyses were applied in all included studies when the associations between frailty and TFS of patients with cirrhosis were estimated. Factors including age, sex, and scores for hepatic dysfunction, such as the MELD and Child-Pugh scores, were adjusted. All included studies had quality scores between seven and nine stars, indicating good quality (Table 2).

**Table 1:** Characteristics of the included studies.

Study	Location	Design	Diagnosis	Patient number	Mean age (years)	Male (%)	Mean MELD score at baseline	Scale for evaluating frailty	No. of patients with frailty	Median follow-up duration (months)	Variables adjusted
Tapper 2015 [26]	USA	RC	Hospitalized patients with cirrhosis	734	57.3	62	17.9	ADL	204	3	Age, sex, active cirrhotic decompensation, HCC, and infection
Lai 2019 [27]	USA	PC	Patients with cirrhosis but no HCC evaluated for transplant	1044	57	57	18	LFI	265	12.5	Age, sex, race, MELD score, ascites, and albumin level
Deng 2020 [28]	China	PC	Patients with cirrhosis	158	64	82	11	CFI	39	24	Age, sex, MELD score, and lymphocyte monocyte ratio
Kremer 2020 [29]	Germany	PC	Outpatients with liver cirrhosis	200	60	56.5	10	CFS	21	12	Age, sex, BMI, alcoholic liver disease, MELD score, ascites, and albumin level
Soto 2021 [36]	Chile	PC	Patients with cirrhosis	126	64	52.4	15	FFI	82	29	Age, sex, MELD score, CPS, Hb, Albumin, sodium, SCr, PLT, and HE, EV, ascites, and HCC
Lin 2021 [30]	USA	PC	Patients with cirrhosis evaluated for transplant	517	61	59	12	LFI	124	8.8	Age, sex, albumin level and MELD score
Skladany 2021 [35]	Slovakia	PC	Hospitalized patients with cirrhosis	385	58.1	62.4	16.8	LFI	184	6.7	Age, sex, MELD score, CRP, HCC, and etiology of cirrhosis
Román 2021 [32]	Spain	PC	Patients with cirrhosis	135	62.7	71.9	9.8	FFI	35	33	Age, sex, and MELD score
Serper 2021 [33]	USA	PC	Patients hospitalized with cirrhosis complications	211	57	55	21	LFI	124	8.3	Age, sex, and MELD score
Mahmud 2021 [31]	USA	RC	Patients hospitalized with cirrhosis for diverse surgeries	804	63	96.6	9	HFRS	390	6	Age, sex, race, etiology of liver disease, ASA score, sodium, SCr, total bilirubin, and PLT
Siramolpiwat 2021 [34]	Thailand	PC	Patients with compensated cirrhosis	152	62.5	57.3	9.2	LFI	37	14.9	Age, sex, MELD score, CPS, bilirubin, albumin, sodium, and PLT
Xu 2021 [37]	USA	RC	Patients with cirrhosis evaluated for transplant	247	57	59	17	LFI	66	8	Age, sex, MELD score, KPS, albumin, and ascites
Lin 2022 [39]	USA	PC	Patients with cirrhosis	116	56	55	15	LFI	26	7.3	Age, sex, and MELD score
Wang 2022 [41]	Canada, USA and India	PC	Patients with cirrhosis	822	55.2	65.8	15.5	LFI	201	14.4	Age, sex, and MELD score
Singh 2022 [40]	India	PC	Patients with cirrhosis	116	50.2	86.2	16	LFI	50	6	Age, sex, MELD score, CPS, BMI, and etiology of liver disease
Guo 2022 [38]	China	RC	Patients hospitalized for decompensated cirrhosis	221	63	46.2	12	CFI	32	24	Age, sex, MELD score, CPS, BMI, sodium, albumin, SCr, infection, ascites, and alcoholic liver disease
Luo 2023 [42]	China	PC	Patients with cirrhosis	285	59.1	51.6	12	FFI	103	19.8	Age, sex, CPS, and CCI

MELD, model for end-stage liver disease; RC, retrospective cohort; PC, prospective cohort; ADL, Activities of Daily Living; LFI, Liver Frailty Index; CFI, Carolina Frailty Index; CFS, Clinical Frailty Scale; FFI, Fried Frailty Index; HFRS, Hospital Frailty Risk Score; HCC, hepatocellular carcinoma; CPS, Child-Pugh Score; Hb, hemoglobin; PLT, platelet count; SCr, serum creatinine; HE, hepatic encephalopathy; BMI, body mass index; CCI, Charlson Comorbidity Index; KPS, Karnofsky Performance Scale; EV, esophageal varices; CRP, C-reactive protein; ASA, American Society of Anesthesiology.

**Table 2:** Study quality evaluation via the Newcastle-Ottawa scale.

Study	Representativeness of the exposed cohort	Selection of the non-exposed cohort	Ascertainment of exposure	Outcome not present at baseline	Control for age and sex	Control for other confounding factors	Assessment of outcome	Enough long follow-up duration	Adequacy of follow-up of cohorts	Total
Tapper 2015 [26]	1	1	1	1	1	0	1	0	1	7
Lai 2019 [27]	1	1	1	1	1	1	1	1	1	9
Deng 2020 [28]	1	1	1	1	1	1	1	1	1	9
Kremer 2020 [29]	1	1	1	1	1	1	1	1	1	9
Soto 2021 [36]	1	1	1	1	1	1	1	1	1	9
Lin 2021 [30]	1	1	1	1	1	1	1	0	1	8
Skladany 2021 [35]	1	1	1	1	1	1	1	0	1	8
Román 2021 [32]	1	1	1	1	1	1	1	1	1	9
Serper 2021 [33]	1	1	1	1	1	1	1	0	1	8
Mahmud 2021 [31]	0	1	1	1	1	1	1	0	1	7
Siramolpiwat 2021 [34]	1	1	1	1	1	1	1	1	1	9
Xu 2021 [37]	0	1	1	1	1	1	1	0	1	7
Lin 2022 [39]	1	1	1	1	1	1	1	0	1	8
Wang 2022 [41]	1	1	1	1	1	1	1	1	1	9
Singh 2022 [40]	1	1	1	1	1	1	1	0	1	8
Guo 2022 [38]	0	1	1	1	1	1	1	1	1	8
Luo 2023 [42]	1	1	1	1	1	1	1	1	1	9

### Meta-analysis results

**Fig 2:**
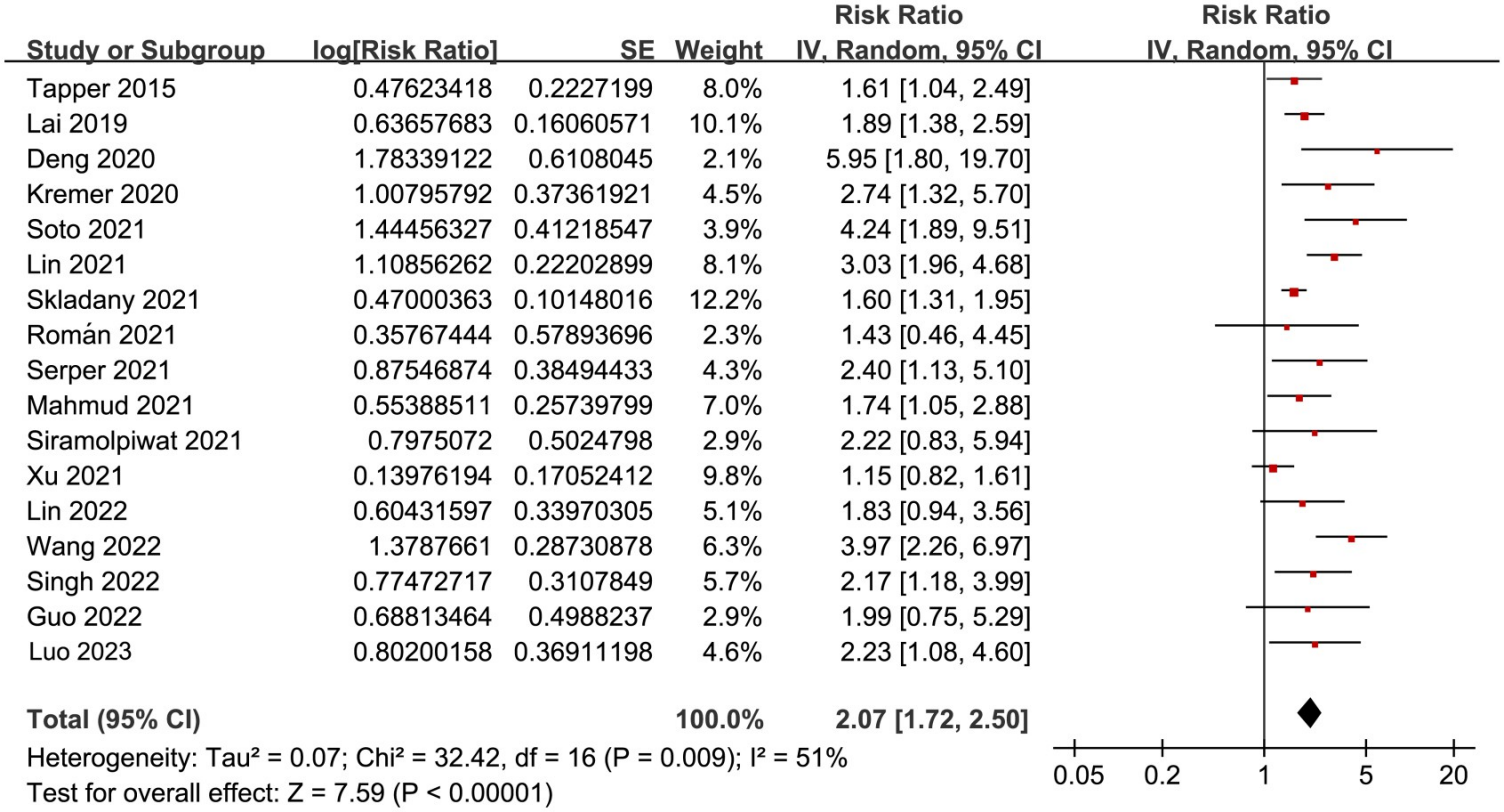
Forest plots for the overall meta-analyses regarding the association between frailty and TFS of patients with liver cirrhosis.

**Fig 3:**
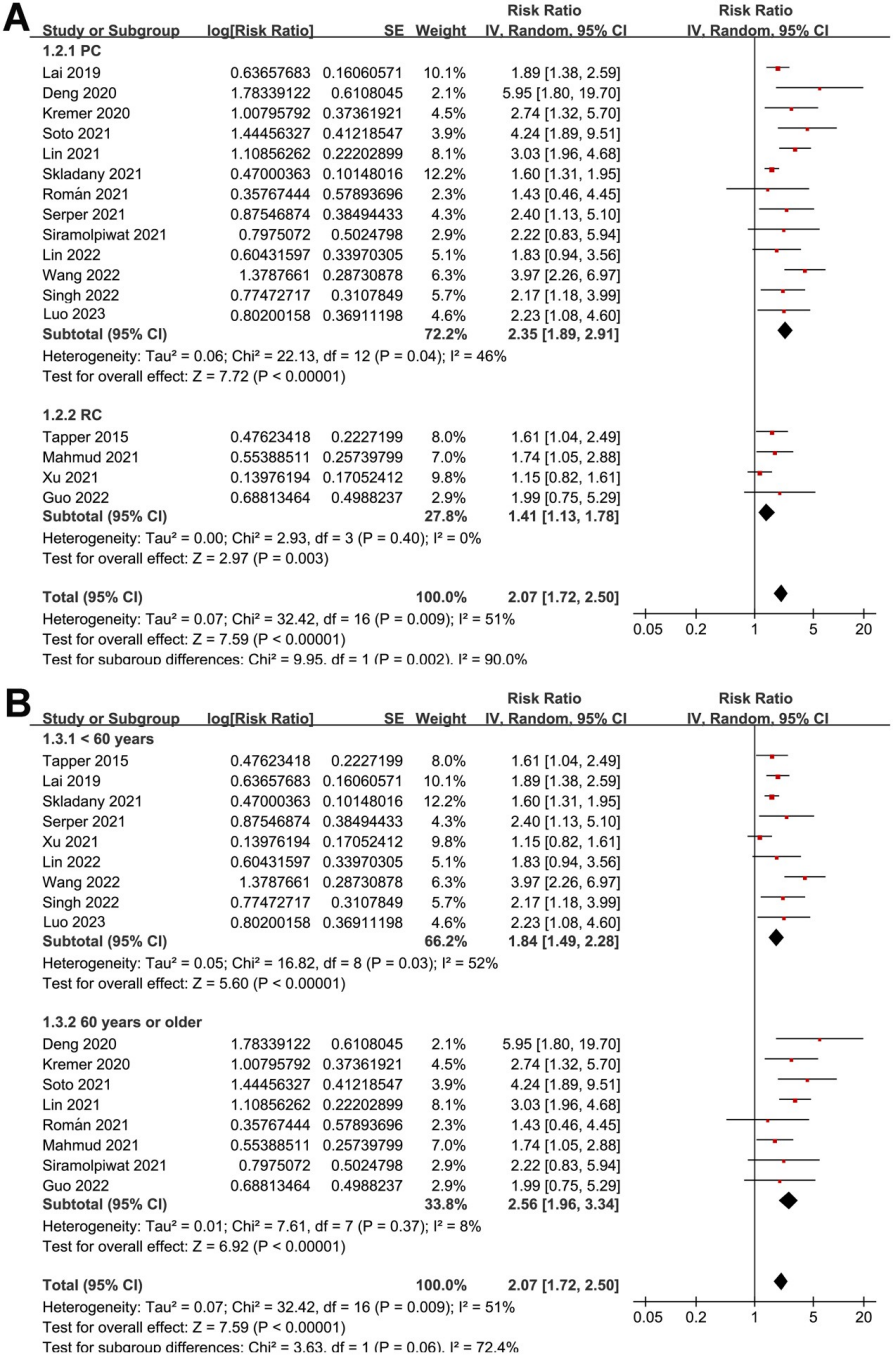
Forest plots for the subgroup analyses regarding the association between frailty and TFS of patients with liver cirrhosis; A, subgroup analysis according to study design; and B, subgroup analysis according to the mean age of the patients.

**Fig 4:**
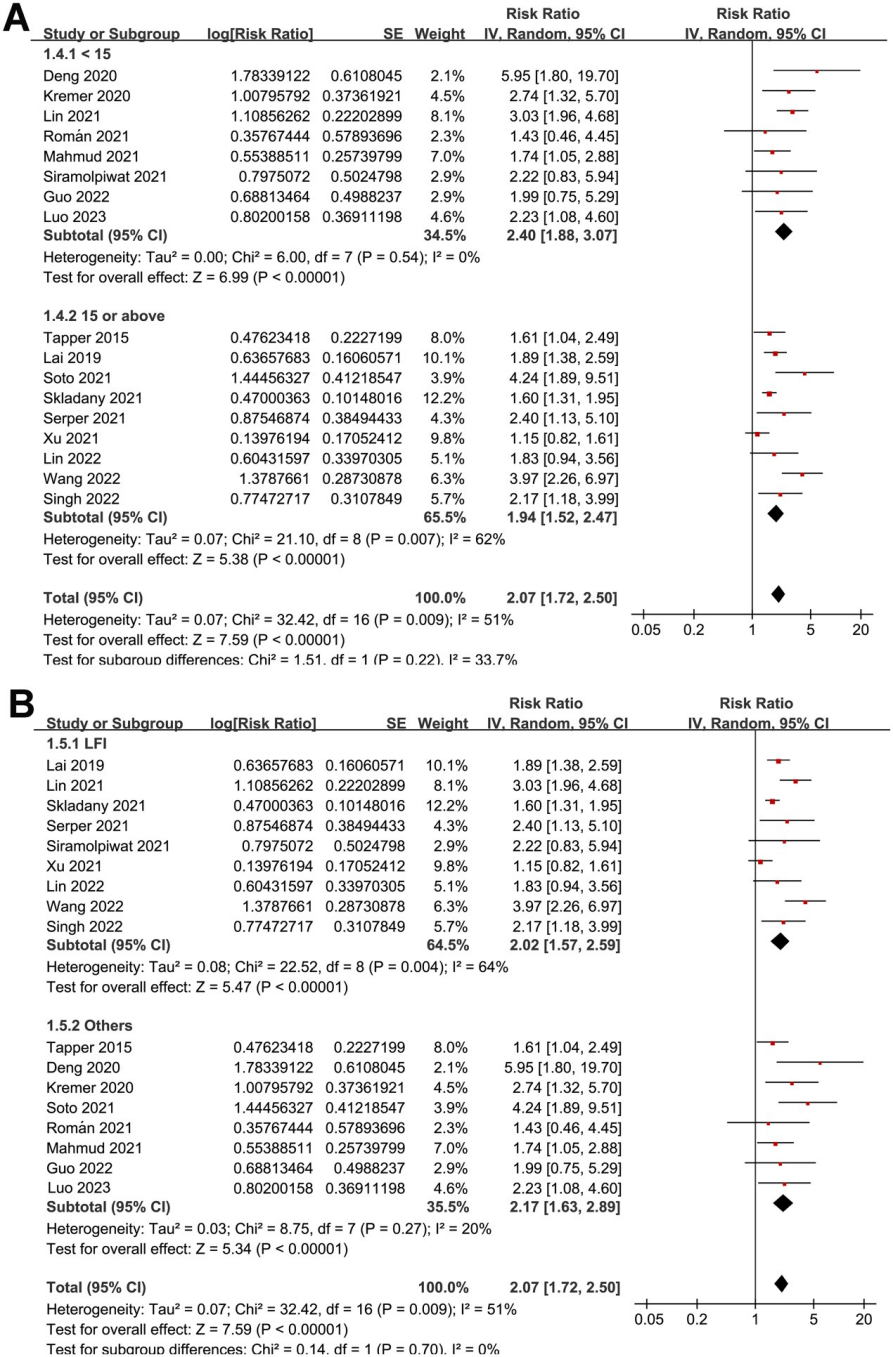
Forest plots for the subgroup analyses regarding the association between frailty and TFS of patients with liver cirrhosis; A, subgroup analysis according to average MELD score at baseline; and B, subgroup analysis according to the scales for frailty evaluation.

**Fig 5:**
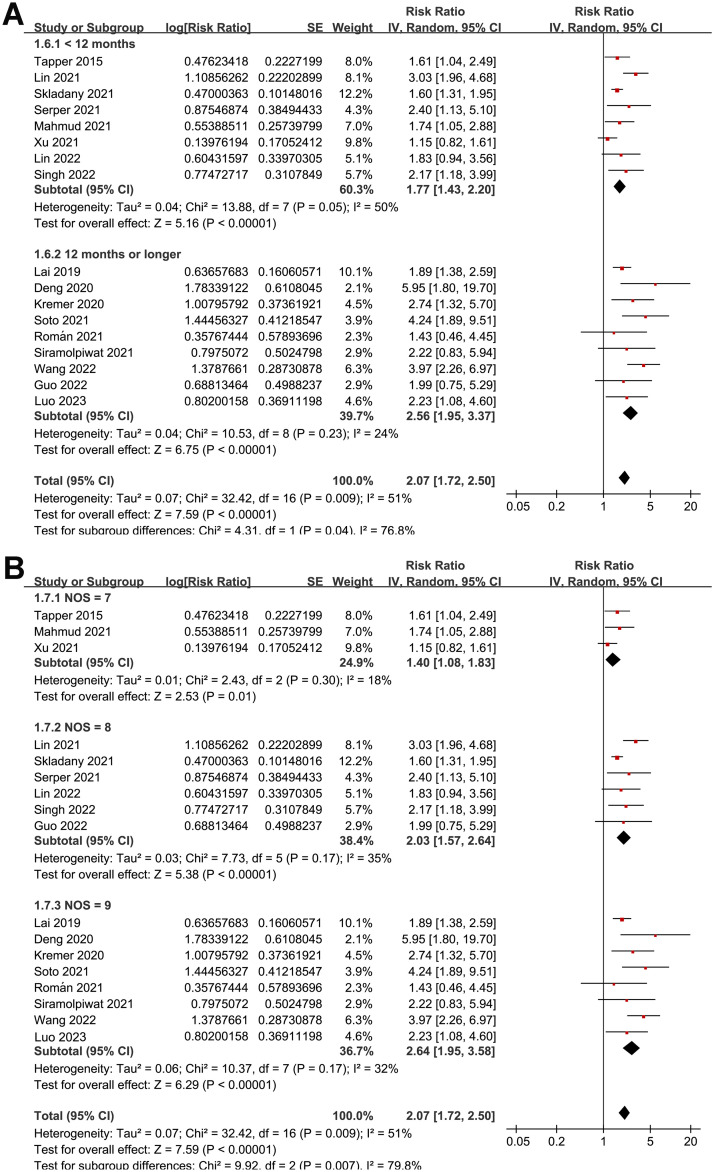
Forest plots for the subgroup analyses regarding the association between frailty and TFS of patients with liver cirrhosis; A, subgroup analysis according to follow-up durations; and B, subgroup analysis according to study quality scores.

Overall, pooled results of 17 studies [26–42] showed that frailty was associated with a poor TFS (RR: 2.07, 95% CI: 1.72 to 2.50, *p*<0.001) in patients with liver cirrhosis, with moderate heterogeneity (p for Cochrane Q test = 0.009, *I*^*2*^ = 51%; Fig 2). In addition, sensitivity analysis by omitting one dataset at a time showed consistent results (RR: 1.95 to 2.17, *p* all<0.05). Moreover, results of subgroup analyses according to study design (Fig 3A), mean age of patients (Fig 3B), the average MELD score at baseline (Fig 4A), scales for frailty evaluation (Fig 4B), follow-up duration (Fig 5A), and study quality score (Fig 5B) also showed a consistent association between frailty and TFS (*p* for each subgroup effect all<0.05). Specifically, the association between frailty and poor TFS seemed to be stronger in prospective studies as compared to retrospective studies (RR: 2.35 versus 1.41, *p* for subgroup difference = 0.002; Fig 3A), in studies with follow-up duration ≥12 months compared to those <12 years (RR: 2.56 versus 1.77, p for subgroup difference = 0.04; Fig 5A), and in studies with NOS = 9 or 8 compared to those with NOS = 7 (RR: 2.64 and 2.03 versus 1.40, *p* for subgroup difference = 0.007; Fig 5B).

### Publication bias

**Fig 6:**
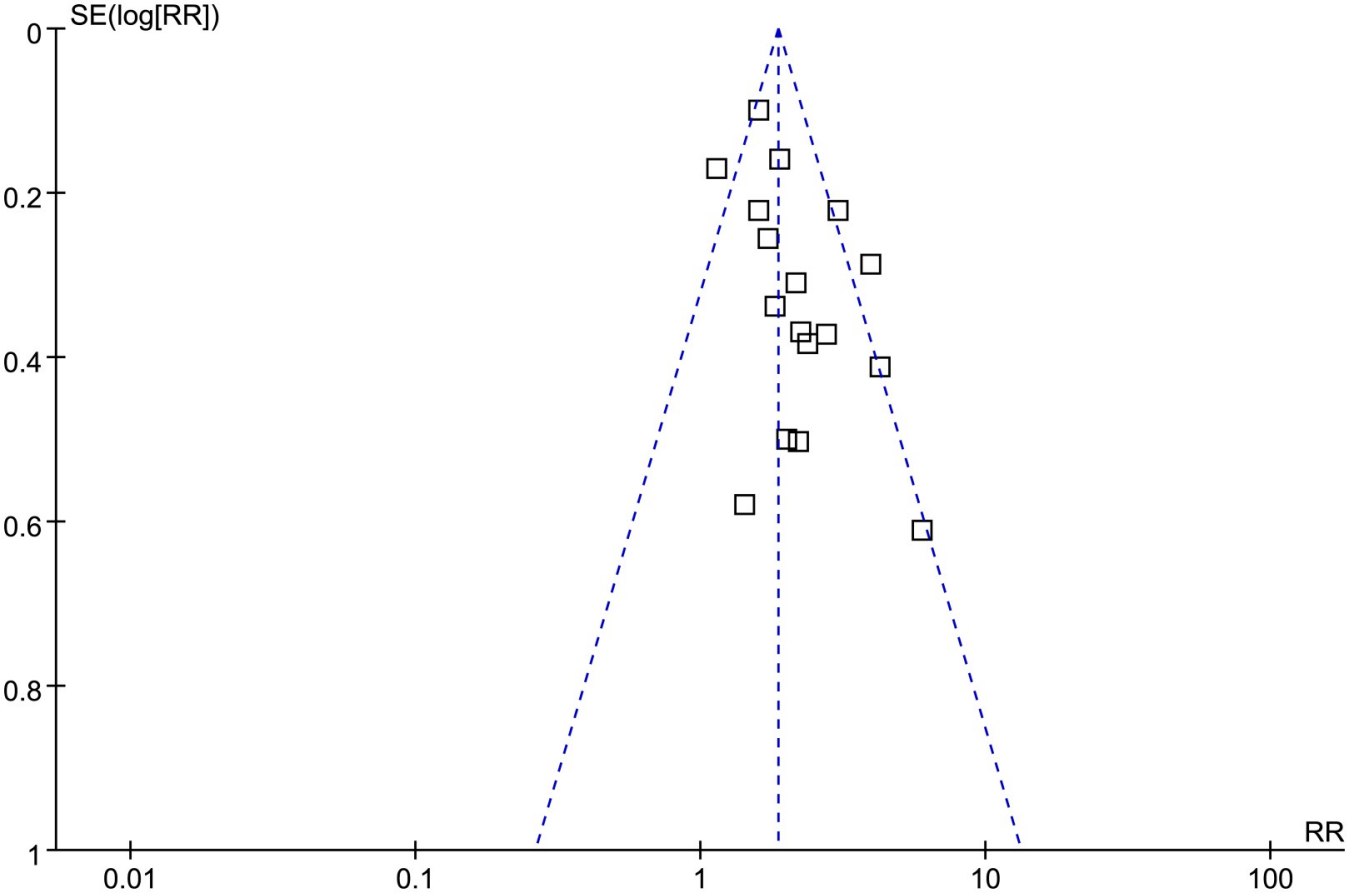
Funnel plots for the publication bias underlying the meta-analysis regarding the association between frailty and TFS of patients with liver cirrhosis.

The funnel plots for the meta-analysis of the association between frailty and TFS in patients with cirrhosis are shown in Fig 6. Based on visual examination, the plots are symmetrical, suggesting low publication bias. Additionally, Egger’s regression tests indicated a low likelihood of publication bias (*p* = 0.39).

## Discussion

In this systematic review and meta-analysis, we combined the findings from 17 pertinent cohort studies, uncovering that cirrhotic patients with frailty exhibited notably poorer TFS than their non-frail counterparts over a follow-up period spanning 3 to 29 months. The consistent results fortify the credibility of this conclusion yielded through rigorous sensitivity analyses and well-considered subgroup analyses. These latter analyses considered study design, mean patient age, average MELD score at baseline, the scale used for evaluating frailty, follow-up duration, and study quality scores. The evidence amassed strongly indicates that frailty may serve as a significant predictor of TFS in patients with liver cirrhosis.

The mechanisms underlying the association between frailty and a poor TFS of patients with cirrhosis may be multifactorial. A previous study including 587 patients with cirrhosis listed for liver transplantation showed that patients with frailty had significantly increased days of hospitalization and a higher incidence of infection [43], which was independent of the severity of cirrhosis as evaluated by the MELD score. Similarly, another study showed that frailty measured by gait speed was a strong risk factor for hospitalization for all cirrhosis complications, which also explained the association between frailty and poor prognosis of patients with cirrhosis [44]. In addition, a recent study showed that frailty might be a risk factor for in-hospital mortality of patients with cirrhosis undergoing transjugular intrahepatic portosystemic shunt [45]. These findings also suggested that cirrhotic patients with frailty may be at high risk for receiving invasive treatments, which may be another potential reason for the poor long-term clinical outcomes of these patients [45]. Pathophysiologically, frailty has been related to upregulated inflammatory response, impaired systemic immune dysfunction, and gut microbiota disorder [46–48], which may also partly explain the association between frailty and poor TFS in patients with cirrhosis. Studies are warranted to determine the molecular pathways involved.

Results of the subgroup analyses generally suggested a consistent relationship between frailty and poor TFS of patients with cirrhosis in multiple predefined clinical conditions. As for the subgroup interaction, we found that the association between frailty and poor TFS may be more remarkable in prospective studies and studies with a high NOS. These results further validated the reliability of the results, primarily driven by high-quality studies. In addition, we found a trend toward a stronger association between frailty and poor TFS in older patients (p for subgroup difference = 0.06) and a more remarkable association in studies with follow-up over 12 months. These findings suggested that using frailty as a predictor of poor TFS may be more suitable for evaluating the long-term prognosis of older patients with cirrhosis. Although no between-subgroup difference was observed according to the different evaluating scales for frailty, LFI was mostly used among the included studies. The parameter in question was initially proposed by Lai et al. in 2017 and determined by evaluating three performance metrics: handgrip strength, chair stands, and balance [49]. It is worth noting that the reproducibility of LFI has been confirmed by various researchers, thus endorsing its utilization as a reliable indicator of frailty in cirrhosis patients [50]. In addition to its potential prognostic value in patients with cirrhosis, there is evidence to suggest that improvement of frailty in these patients may be associated with survival benefits [5,30]. However, these findings should be validated in large-scale clinical trials.

The methodological strengths of this study encompass an exhaustive database search to procure the most recent literature, the inclusion of cohort studies to establish a longitudinal relationship between frailty and poor TFS, the amalgamation of data from multivariate analyses to mitigate the effects of confounding factors, and the application of multiple sensitivity and subgroup analyses to underscore the robustness of the findings. Nevertheless, the meta-analysis is not without limitations. Firstly, the approaches to evaluating frailty diverged among the studies included. Though the LFI was predominantly utilized, the optimal scale and threshold for appraising frailty in cirrhosis patients still warrant determination. Secondly, as a synthesis of observational studies, it is conceivable that unadjusted residual factors might still confound the association between frailty and poor TFS of cirrhosis patients. Moreover, the follow-up durations of the included studies were relatively short (average: 11.5 months). The potential efficacy of frailty for the prediction of long-term mortality in patients with cirrhosis should be validated in large-scale prospective studies in the future. Additionally, the impact of varying cirrhosis etiologies on the relationship between frailty and poor TFS remains unexplored. Besides, we only investigated the relationship between frailty and TFS in patients with cirrhosis. Studies are needed to determine the association between frailty and the risk of other complications in patients with cirrhosis, such as the incidence of hepatic encephalopathy. Finally, a causal link between frailty and adverse clinical outcomes in cirrhosis cannot be inferred from this meta-analysis, as it only included observational studies and only observed the outcome of liver transplant-free mortality. Clinical trials would be instrumental in assessing whether ameliorating frailty could translate into survival benefits for these patients.

## Conclusion

In conclusion, the findings of this systematic review and meta-analysis suggest that frailty may serve as a significant predictor of liver transplant-free mortality in patients with cirrhosis. These insights underscore the importance of integrating frailty assessment into the clinical care protocols for cirrhosis patients. Ongoing research and concerted efforts are essential to formulate the optimal scale for evaluating frailty in this patient population and explore whether interventions to improve frailty could be associated with a reduced risk of liver transplant-free mortality.

## Supporting information

S1 Checklist PRISMA 2009 checklist. (DOC)
